# Hyaluronic High Definition Fill Technique

**DOI:** 10.1111/jocd.16692

**Published:** 2024-11-17

**Authors:** Luciana M. Lourenço, David Di Sessa, Ana Carolina Franco Tebet, Maria Gabriela Ortiz de Noronha, Helena Lourenço de Medeiros, Rosa Sigrist

**Affiliations:** ^1^ Private Practice in Dermatology São Paulo Brazil; ^2^ Faculty Ciências Médicas de Minas Gerais Belo Horizonte Brazil; ^3^ Department of Radiology, Hospital das Clínicas–Faculty of Medicine University of São Paulo Sao Paulo Brazil

**Keywords:** body image, dermal filler, hyaluronic acid

## Abstract

**Introduction:**

Achieving a defined abdomen through traditional surgical methods like liposuction and abdominoplasty involves risks and significant downtime. Hyaluronic acid (HA) body filling has emerged as a minimally invasive alternative, offering immediate results with reduced recovery periods.

**Patients and Methods:**

The high‐definition abdomen through HA technique (HHD) was implemented on young men with BMI < 24.9, excluding those with obesity or excessive skin laxity. HA gel with large particle sizes (800–1800 μm) and high G prime was chosen based on subcutaneous thickness. Injections were guided by ultrasound into the lamellar fat layer between Camper's fascia and Scarpa's fascia, targeting abdominal metameres for enhancement. Participants completed the Global Aesthetic Improvement Scale.

**Results:**

The technique produced satisfactory outcomes, enhancing abdominal metamere definition. Mild post‐procedural hematomas and transient discomfort were noted. At 10 months, 71.4% of patients reported “much improved,” and 28.6% reported “improved,” per the GAIS questionnaire.

**Discussion:**

Customizing HA characteristics and employing ultrasound guidance ensured safe and precise injections, minimizing vascular risks. Limitations such as HA's temporary nature and exclusions for surgical candidates were acknowledged.

**Conclusion:**

HHD using large particle HA proved effective in achieving aesthetic abdominal enhancements with minimal risks and downtime. This technique not only provided immediate results but also contributed to enhanced patient satisfaction and self‐esteem. Further research is needed to explore broader applications and refine outcomes across diverse patient groups.

## 1 Introduction

The defined abdomen has been one of the main goals for patients seeking plastic surgery for many years. Among the main procedures, liposuction is one of the most commonly used for this purpose. The technique involves using suction cannulas to remove fat deposits in patients with localized fat. Its disadvantages include the risk of complications such as contour irregularities, thromboembolism, and downtime [1, 2]. Another technique is abdominoplasty, which is the method of choice for patients with excess skin after significant weight loss, post‐pregnancy, and abdominal flaccidity in general [3]. Its main disadvantages are the scar from the removal of excess skin and the postoperative time [4].

With the advancement of technology and science, it is increasingly possible to achieve desired body contour with great safety, low downtime, and immediate results [1, 5, 6, 7] through minimally invasive procedures using injectables.

More recently, body contouring with hyaluronic acid (HA) has emerged as an excellent alternative to surgical methods in achieving the desired shape [13]. This minimally invasive technique involves the injection of HA, a biocompatible and absorbable substance, into specific body areas to enhance volume and definition. Initial technique descriptions focused on the buttocks, and more recently, applications have extended to the abdomen [6, 8, 9, 10].

Understanding anatomy is essential for a safe and accurate approach. The abdominal fat compartment is composed of deposits located between the muscles and the skin [11]. The superficial fat is called areolar and is separated by Camper's fascia from the deeper fat, the lamellar, which rests on Scarpa's fascia. This latter layer of fat has a significant influence on the appearance of the abdominal contour and is a safe plane for HA injection due to the absence of large vessels or noble structures in the region [12].

Abdominal metameres contribute to the beauty of the abdomen, and their marking is essential for a good result of the technique. Metameres are the individual muscular segments formed by the rectus abdominis muscles and their tendinous insertions. There are three to four tendinous inscriptions in the abdomen, all adherent to the anterior sheath of the rectus and separated by the linea alba, and their distribution is unique in each patient [13, 14].

In this article, we will describe the HHD technique, high‐definition abdomen through body HA, to enhance the abdominal metameres, providing more beauty to the athletic abdomen.

## 2 Materials and Methods

The authors describe a technique with a specific product in a safe plan to increase the demarcation of abdominal metameres for aesthetic purposes in young male patients.

Inclusion criteria were men with a BMI lower than 24.9 seeking to improve abdominal muscle definition.

Exclusion criteria were obesity, excessive muscle and skin flaccidity, local infections, previous hip prosthesis, allergy to HA or known contraindications for filling with HA.

The study was conducted in accordance with the Declaration of Helsinki, and all patients provided informed consent.

**FIGURE 1:**
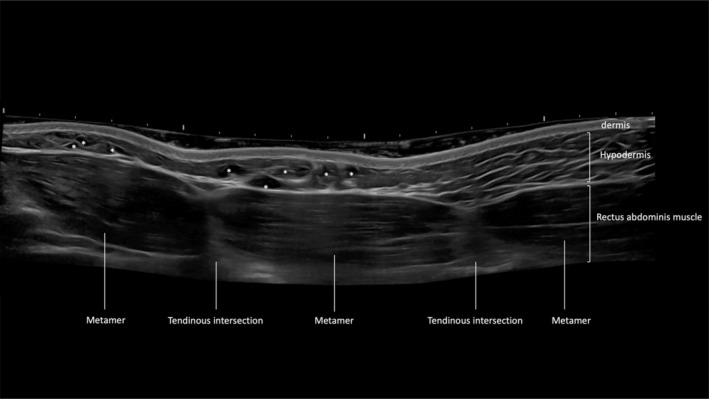
B‐mode ultrasound image of the abdomen (longitudinal view) showing the dermis, hyaluronic acid deposits (*) volumizing the hypodermis, the rectus abdominis muscle divided by the tendinous intersections in metameres.

The product chosen for this technique was a large particle HA gel. Two types of products were used, depending on the patient's skin and subcutaneous thickness. For patients with thicker skin and a thicker subcutaneous layer, the product chosen was HA with larger particles (800–1800 μm) and high G prime (G') of 550 pa presented in 10 and 20 mL syringes (Sofiderm SubSkin). In patients with thinner skin and less thick subcutaneous tissue, the product chosen was with larger particles, slightly smaller than the previous one (1500 μm), however with the same G' and 10 and 20 mL syringes (Sofiderm Derm Plus). The cannula used was the 18G × 70 mm cannula. The syringe used was 3 mL. The chosen plane was the lamellar fat between Camper's fascia and Scarpa's fascia. The application was ultrasound‐guided with a LOGIQ E10 system (GE Healthcare, Waukesha, WI), with a linear probe that ranges from 6 to 24 MHz (Figure 1).

**FIGURE 2:**
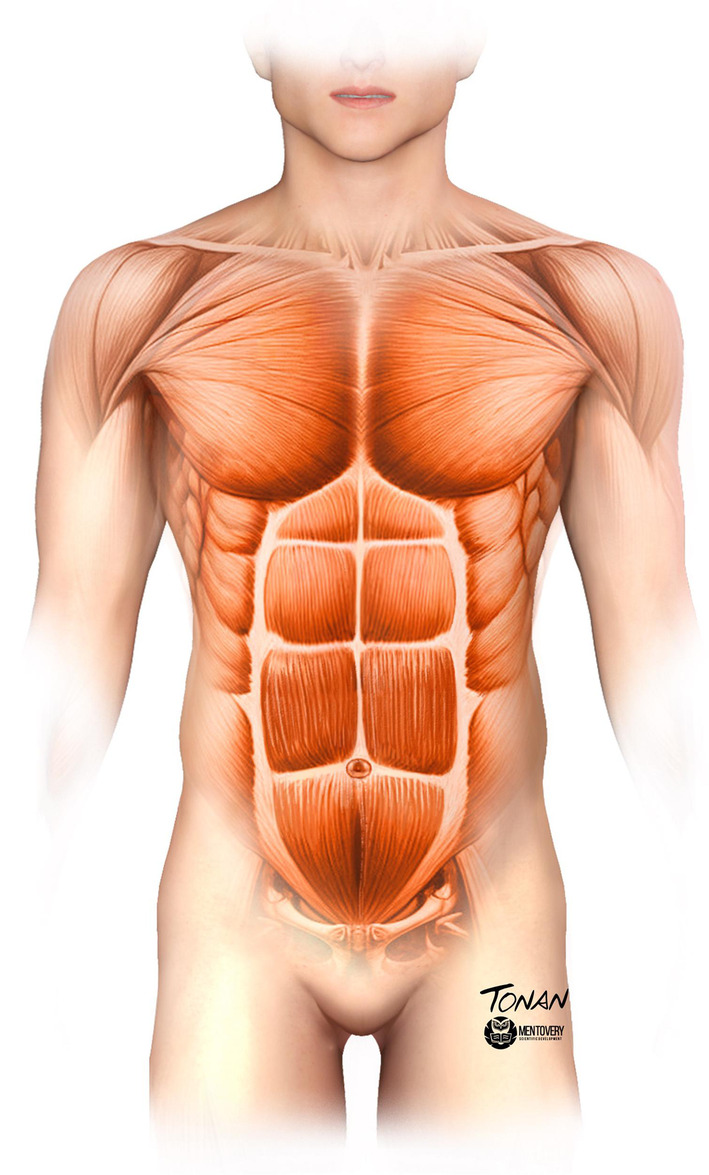
Abdominal division into metameres.

It is important to mark the technique and must be done standing up. The authors divided the abdomen into six metameres (sometimes four, as we do not always treat all metameres) above the navel, three on the right and three on the left, and three inverted triangles that run from the iliac spine toward the groin (Figure 2). Ultrasound was used to check the demarcation of the metameres with the patient standing.

**FIGURE 3:**
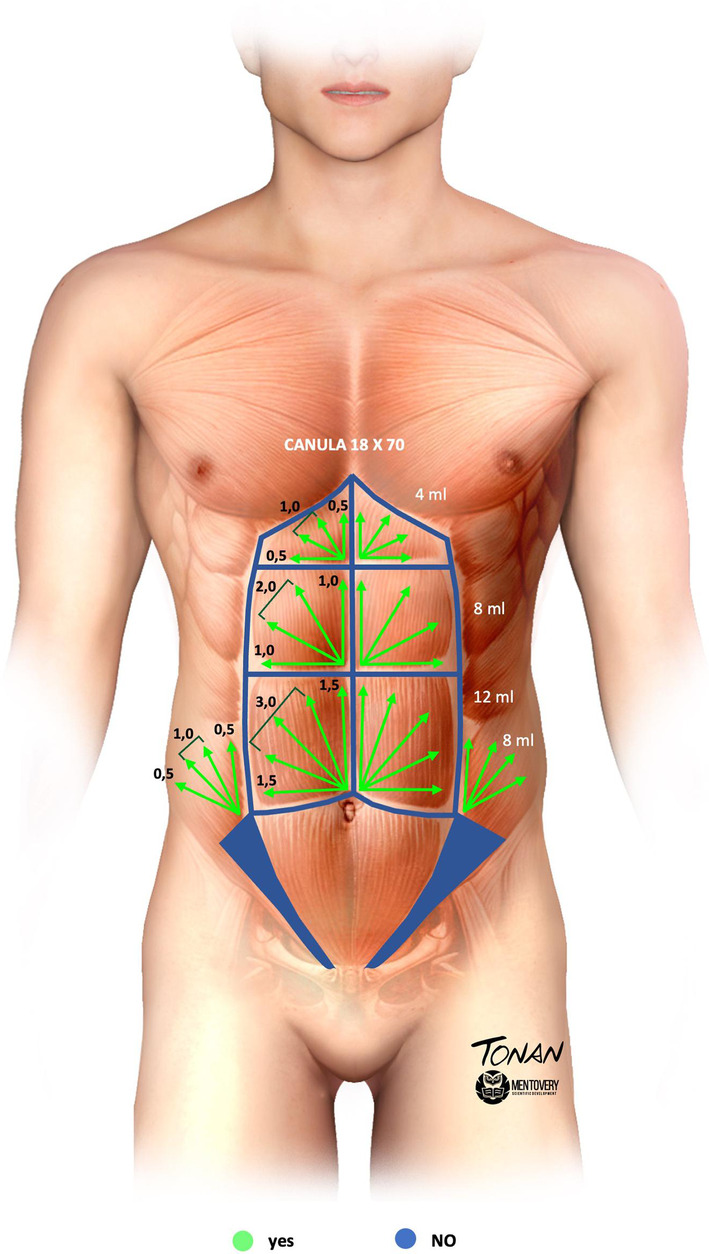
Hyaluronic high definition fill technique representation: In the figure above, two arms of injection were applied in a triangular frame and two to four arms in the central region. This pattern is also applied in the lower inverted triangles.

In the six metameres, the application was carried out with the patient lying down in a “Fan technique” (fan, retroinjection) with an 18 × 70 g cannula, starting from the central part in a cross, in the metameres above the navel from top to bottom and in the others from bottom to top. The authors opted for two arms in the triangle frame and two to four arms in the central region, always in a fan shape. The sum of product of the two arms of the framed region always remained at the same amount of product as the sum of the arms of the fan in the central region, therefore the quantity of product was always greater in the frames. In the lower inverted triangles the same rule is also followed. (Figure 3) The suggested amount depends on the amount of subcutaneous fat and muscular architecture, but it was suggested to be around 20–40 mL in total, varying according to the individual patient's needs, for one treatment session as in Figure 3. In the patients described in this technique, a total of 32 mL of HA was used.

On the day of the procedure, azithromycin 1 g was used (prophylactic dose). The result was evaluated after one session. If there was a need or desire, the patient could repeat the session after 15 days. The patient was instructed not to do physical exercise for 2–3 days. Participants also completed the Global Aesthetic Improvement Scale (GAIS) and were followed for 10 months.

## 3 Results

**FIGURE 4:**
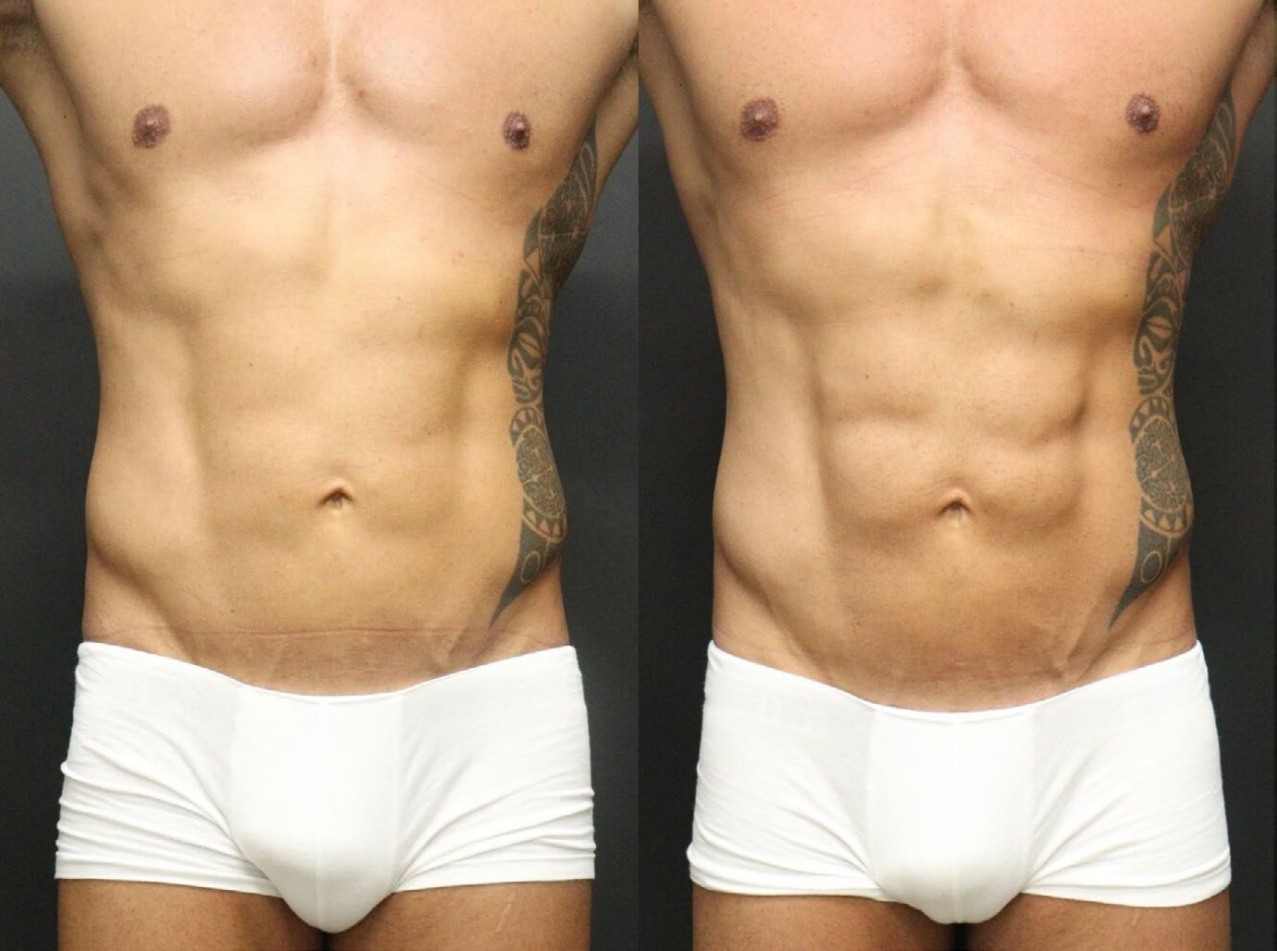
Frontal vision prior and after the treatment with HA filler. It is possible to observe better demarcation and prominent abdominal metameres after the procedure.

**FIGURE 5:**
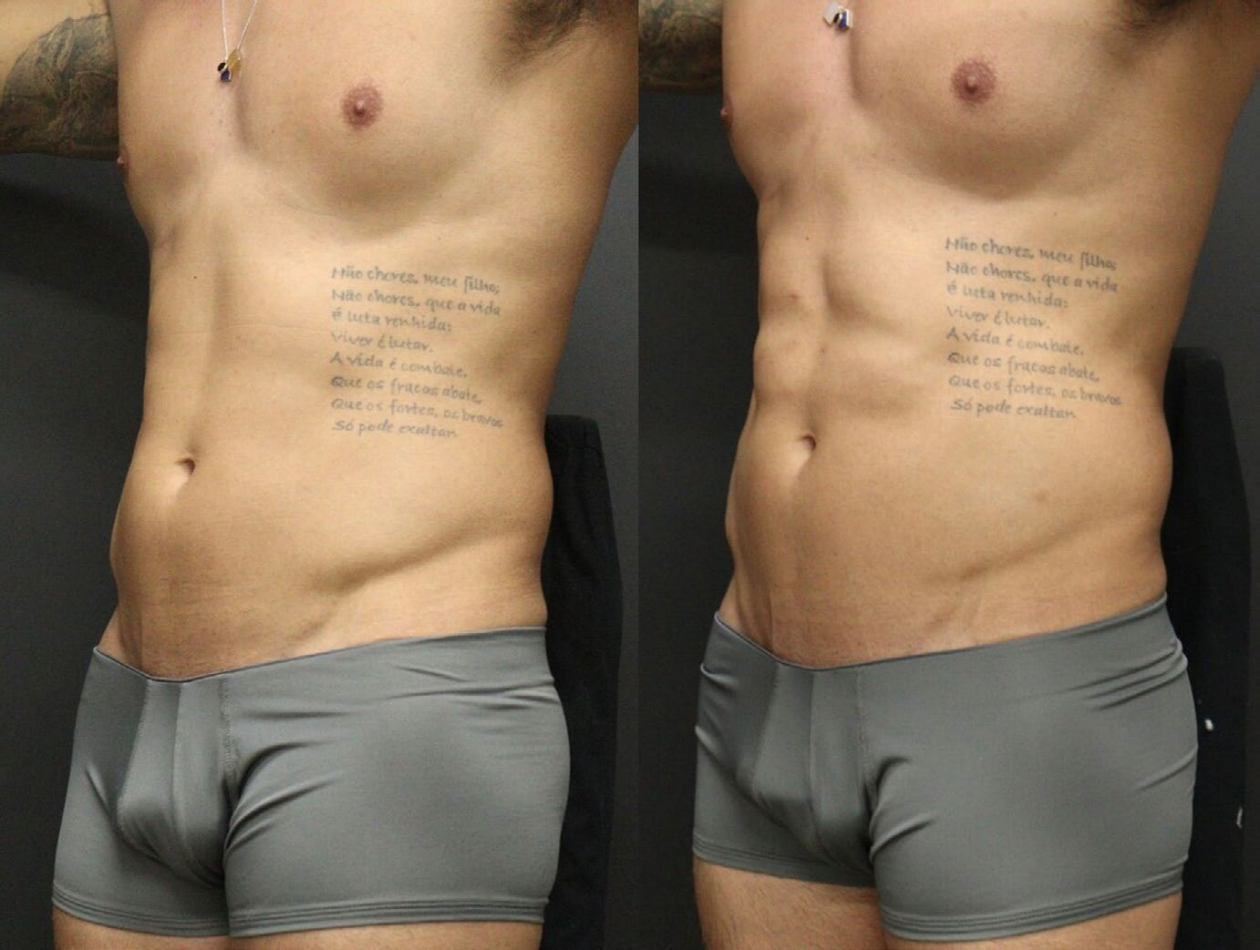
Lateral vision prior and after the treatment with HA filler. There is noticeable improvement in the delineation and prominence of abdominal segments.

The authors report good aesthetic results with the proposed technique (Figures 4 and 5). Prior to treatment, there was evident preservation of musculature with minimal adipose tissue, but absence of muscular definition. It is notable that after one session with HA filler it was possible to achieve a high abdomen definition.

Mild hematomas were noted after the procedure, along with mild pain at the site for up to 3 days. No other complications were observed.

All the 7 patients completed the satisfaction questionnaire (GAIS). After 9 months, 5 patients (71, 4%) reported that their treated areas had “much improved,” and 2 patients (28, 5%) stated that their treated areas had “improved.” Patients reported a high degree of satisfaction with the improvement in quality of life.

## 4 Discussion

The thinner the fat planes, the better the results. The two main indications of the technique are lean patients struggling to achieve abdominal muscle definition through muscle hypertrophy and individuals who already have some muscle definition but desire a more robust appearance of the abdominal musculature.

To achieve this result, the rheology of the product is a determining factor. It is important for the body HA to have a high G' and large particle size, which accounts for resistance to deformation, high lifting power, and result durability [6, 8]. In this study, we used two types of HA, both with very high G', but different particle sizes. For patients with thinner adipose tissue, we opted for HA with a particle size of 1500 μm to prevent the product from being apparent or palpable; for others, we used HA with particles up to 1800 μm. Although both are large particle HA, choosing the ideal product delivers more natural results.

Our technique was performed only on men in this article; however, it can be replicated in women. We suggest using HA with a smaller particle size due to the thickness of female skin.

The studied technique offers a lot of safety, from product selection to application plan. HA, besides being biocompatible and absorbable, has an antidote. If deposited in the wrong place, product accumulation, or a rare vascular involvement occurs, it can be easily dissolved with hyaluronidase, even with high G' and large particle size [7, 14].

The subcutaneous tissue was chosen as the HA application plane in the abdomen, especially above the umbilical line, due to the rarity of large blood vessels above or other risky structures.

The injections are performed superficial to Scarpa's fascia, avoiding possible interactions with intra‐abdominal organs, as well as larger vessels [12, 15]. In cases of anatomical variation, the inferior epigastric artery and vein may be present in the targeted area; however, these vessels usually run parallel anteriorly to the semilunar line bilaterally and end their course below the umbilical scar. This anatomy favors us as injections are not performed anteriorly to the abdominal tendon insertions and are generally performed above the umbilical scar in the central plane of the abdomen [12, 15].

As studied by Lourenço et al., in addition to safety, the subcutaneous tissue allows for better use of the product, which is very important to enable the techniques, as the volume of the products is greater than on the face [6]. This plane is superficial enough for the product to volumize and deep enough not to be palpable.

Regarding the chosen plane, injection in fans allows for a greater spread of the product, leaving the surface more regular [6]. The authors chose to use more product in the frames to better mark the transition line of the metameres.

Although a total of 32 mL of HA was used for each patient in the study, this amount can vary depending on the individual's needs and preferences.

Hyaluronic acid fillers have been successfully utilized in body contouring procedures, including the buttocks. Unlike abdominal applications, where product selection varies according to skin thickness, buttock augmentation consistently employs fillers with higher G' prime and larger particle size. This choice is crucial due to the high‐impact nature and greater fat thickness of the buttocks, which allows for optimal results. The studies by Lourenço et al. provide a valuable comparison to our technique, highlighting both the versatility of hyaluronic acid and the specific considerations required for different anatomical regions [6, 8].

The use of ultrasound was important for verifying the anatomical boundaries of the metameres after their marking, as well as for determining the injection plane, free of larger vessels. Other studies conducted by our group have also utilized ultrasound to determine the injection plane in the gluteal region in successful techniques [6, 8].

When discussing costs, it is important to note that they are variable and influenced by factors such as the brand of the product and the country of purchase. The cost of this procedure is higher compared to traditional facial fillers due to the larger quantity of product used.

Patients reported a high level of satisfaction, as reflected in their responses on the GAIS questionnaire. As observed by Papadopulos NA et al. in patients aiming to treat abdominal definition, these individuals seek not only aesthetic procedures but also an improvement in their mental and emotional satisfaction [16]. After the procedure, all patients reported an improvement in quality of life.

**FIGURE 6:**
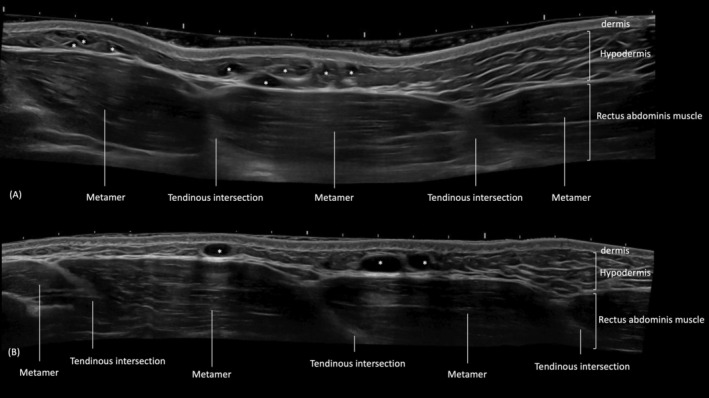
B‐mode ultrasound image of the abdomen (longitudinal view) showing the dermis, hyaluronic acid deposits (*) volumizing the hypodermis, the rectus abdominis muscle divided by the tendinous intersections in metameres. (A) Day of injection. (B) 9 months follow‐up: residual hyaluronic acid deposits remain visible.

After 9 months the patients were re‐evaluated in anti‐ham clinical results and in the ultrasound, hyaluronic acid deposits were found at the applied site, which corroborates the choice of the product used (Figure 6).

The limitations of the technique are as follows: compared to plastic surgery, HA is not permanent; it is a non‐autologous product; cases with surgical indication do not benefit from it.

## 5 Conclusion

The HHD fill technique using HA with large size particles for augmentation of abdomen proved to be a minimally invasive technique providing rapid results and without major risks and downtime. It brought satisfaction to patients, with the improvement in their self‐esteem and quality.

## Conflicts of Interest

The authors declare no conflicts of interest.

## Data Availability

The data that supports the findings of this study are available in the Supporting Information of this article.
